# A rare case of a giant retroperitoneal lipoma with multiple limb and trunk lipomata without familial multiple lipomatosis

**DOI:** 10.1093/jscr/rjac121

**Published:** 2022-03-26

**Authors:** Jason R Laurens, Adam J Frankel, Bernard M Smithers, Geoffrey Strutton

**Affiliations:** Upper Gastro-intestinal and Soft Tissue Unit, Princess Alexandra Hospital, Woolloongabba, QLD, Australia; C/O Department of Surgical Specialties, Princess Alexandra Hospital, Woolloongabba, QLD, Australia; Upper Gastro-intestinal and Soft Tissue Unit, Princess Alexandra Hospital, Woolloongabba, QLD, Australia; Upper Gastro-intestinal and Soft Tissue Unit, Princess Alexandra Hospital, Woolloongabba, QLD, Australia; Mayne Professor and Head, Discipline of Surgery, The University of Queensland, Woolloongabba, QLD, Australia; Department of Anatomical Pathology, Princess Alexandra Hospital, Woolloongabba, QLD, Australia

## Abstract

Retroperitoneal lipoma is exceedingly rare, and due to the difficulty in distinguishing between retroperitoneal lipoma and well-differentiated liposarcoma (WDLS), recommendation is en-bloc resection. A 58-year-old male was investigated for scrotal swelling, ultrasound and computed tomography showed a well-defined lipomatous mass occupying much of the left side of the lower abdomen. At laparotomy, a large left-sided retroperitoneal mass was found. Histology reported a 160 mm × 150 mm × 90 mm fatty tumour weighing 1540 g. MDM2 gene amplification was not present on fluorescence in situ hybridization. No significant somatic signatures were identified on whole exome sequencing. Retroperitoneal fatty tumours represent a diagnostic dilemma. Sampling via core biopsy has been recorded at 85% accuracy for WDLS. Positive amplification of the MDM2 gene supports a diagnosis of WDLS; however, a negative biopsy does not exclude the diagnosis due to varied amplification among different cells in the same tumour.

## INTRODUCTION

Retroperitoneal lipoma is exceedingly rare, and due to the difficulty in distinguishing between retroperitoneal lipoma and well-differentiated liposarcoma, the treatment recommendation is en-bloc resection. We report the rare and unusual case of giant retroperitoneal lipoma in association with multiple limb and trunk lipoma.

## CASE REPORT

**Figure 1:**
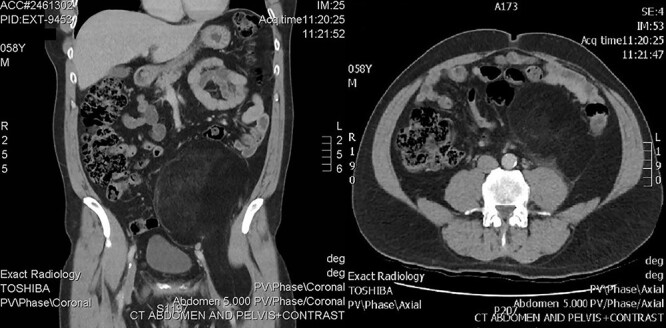
CT showing retroperitoneal mass.

A 58-year-old male presented to his general practitioner with right scrotal swelling that developed over a week. He had an ultrasound followed by a computed tomography (CT) scan, which demonstrated a right hydrocoele, but also showed a well-defined lipomatous mass occupying much of the left side of the lower abdomen, extending from the edge of Gerota’s fascia behind the inguinal ligament towards the lesser trochanter. It was posterolateral to the external iliac artery and vein as they exited the pelvis. There was mild dilatation of the left upper ureter likely due to mass effect, but the left pararenal fat did not appear involved in the mass (Fig. 1). Subtle heterogeneity was noted on the scans which prompted a provisional diagnosis of well-differentiated liposarcoma (WDLS).

He was referred to our sarcoma service. Medical history was dyslipidaemia, four coronary artery stents for ischaemic heart disease, an unprovoked deep vein thrombosis in his left leg 13 years previously, transurethral resection of the prostate for benign prostatic hyperplasia and lumbar discectomy. He was an active smoker (15/day). His medications were clopidogrel, rosuvastatin and perindopril. There was no family history of lipomas. The abdominal mass was easily palpated in his left lower quadrant and had a soft consistency. There was a hydrocele in the right hemiscrotum. Notably, he had $\sim$10 subcutaneous soft, well-circumscribed mobile lipomata up to 5 cm on his trunk and limbs. Full blood picture, including renal and liver function tests were normal.

**Figure 2:**
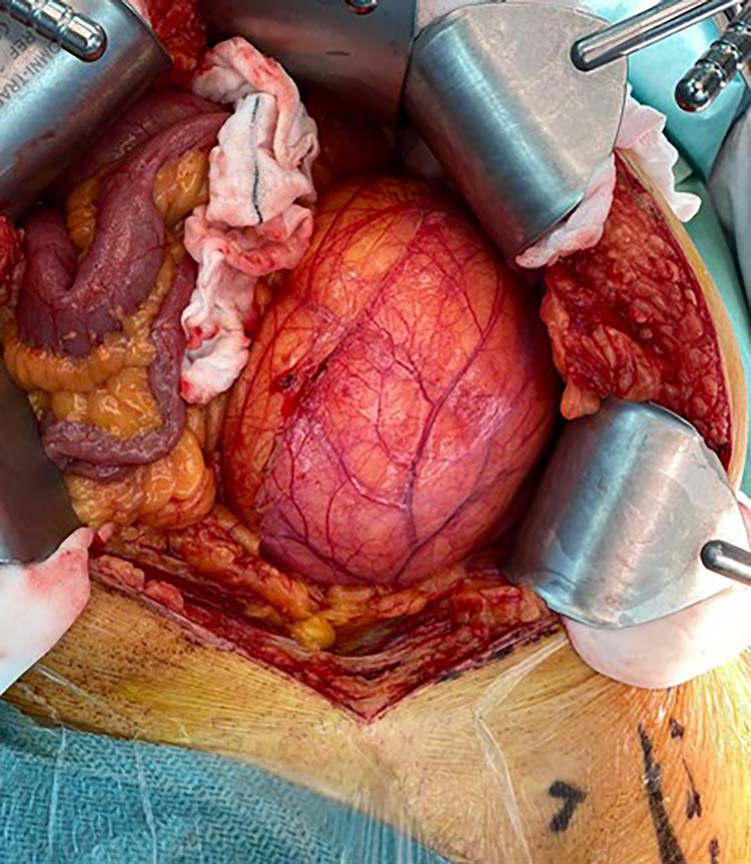
Intraoperative photo showing size of lesion and displacement of bowel.

**Figure 3:**
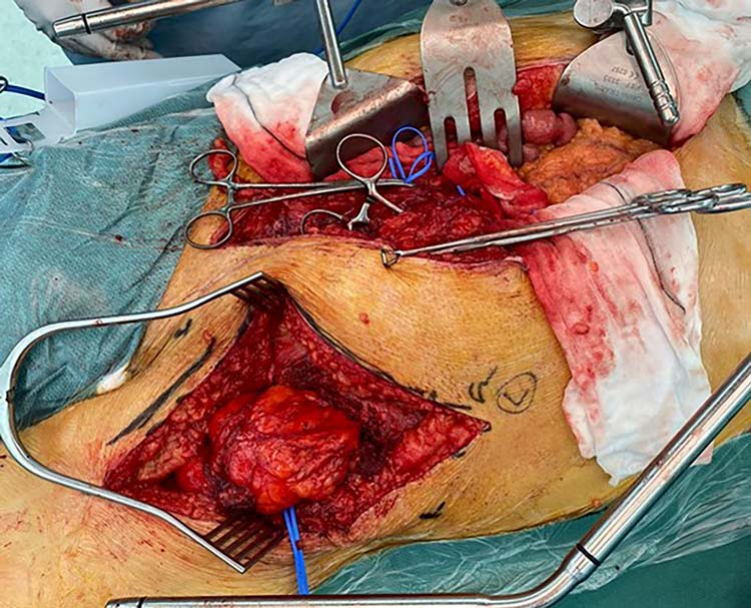
Intraoperative photo showing groin incision with inguinal ligament divided, to allow adequate dissection in lower pelvis and groin.

Following discussion in the sarcoma multi-disciplinary team (MDT) meeting, en-bloc resection was recommended. Although at laparotomy, a large left-sided retroperitoneal mass was found, macroscopically it appeared to be dark yellow to orange adipose tissue within a semitranslucent capsule, involving the psoas and displacing the L2 trunk (Fig. 2). Macroscopically, it did not involve Gerota’s fascia or the mesocolon, allowing kidney and bowel preservation. The left external iliac and common femoral vessels were not involved, and the left femoral nerve was stretched on the anterolateral surface and able to be preserved. A separate incision in the groin was required with division of the inguinal ligament to resect the mass where it was adherent to the psoas insertion at the lesser trochanter (Fig. 3). The tumour was removed en-bloc. A Jaboulay procedure of the right hydrocele was performed. He was discharged postoperative Day 10, as he was mobilizing independently, he was not prescribed chemical deep vein thrombosis (DVT) prophylaxis. His postoperative course was complicated by a superficial thrombosis of the left leg treated with low molecular weight heparin and graduated compression stocking. Although he had a previous unprovoked left leg DVT, and new left leg superficial thrombus, no prothrombotic workup was undertaken due to this thrombus having an identified risk factor, reduced mobility and bed rest. At 6-month review, he had fully recovered.

Histology reported a 160 mm × 150 mm × 90 mm fatty tumour weighing 1540 g. Microscopically, an expert soft tissue pathologist favoured lipoma. MDM2 gene amplification was not present on fluorescence in situ hybridization following testing of multiple sites of the tumour. He underwent whole exome sequencing to further investigate HMGA2 with review at the Queensland Molecular Tumour Board. No significant somatic signatures were identified.

## DISCUSSION

Giant retroperitoneal lipomas are remarkably rare, with 20 cases reported in the English literature [1]. Although there is no consensus which distinguishes giant lipomas from non-giant lipoma, all previously reported cases of giant retroperitoneal lipoma describe tumours with at least one dimension greater than 10 cm [1]. Fatty tumours of the retroperitoneum represent a diagnostic dilemma, due to the difficulty in distinguishing between benign lipoma and liposarcoma, particularly WDLS [2]. CT imaging cannot definitively diagnose benign or malignant adipocytic lesions [3]. Magnetic resonance imaging (MRI) has shown to be useful but imperfect in distinguishing lipomas and WDLS [4]. Radiological features of retroperitoneal lipoma include fat signal attenuation and contain few if any septations, whereas WDLS also demonstrate fat attenuation and inversely commonly contain septa [5]. WDLS often but not always contain mature fatty elements or non-adipose tissue [5, 6]. MRI was not recommended in this case due to the heterogeneity on CT, because although MRI has been reported as 100% specific in the diagnosis of simple lipoma, this is in the setting of homogeneous fatty mass, without muscle fibres, blood vessels, fibrous septa or areas of necrosis or inflammation, which can all confound the correct imaging diagnosis [4]. Although the Retroperitoneal Sarcoma Transatlantic Working Group recommend image-guided percutaneous core biopsy [7], it is important to acknowledge the accuracy of sampling via core biopsy has been recorded to be 85% for WDLS [8, 9]. No studies report core sampling of retroperitoneal lipoma, and there is clearly potential for sampling error with such large tumours. Positive amplification of the MDM2 gene supports a diagnosis of WDLS; however, a negative biopsy does not exclude the diagnosis due to varied amplification among different cells in the same tumour [10]. En bloc resection is the cornerstone of management, which applies equally to WDLS and to large, radiographically ‘benign’ lipomatous masses, although the preservation of specific organs should be considered on an individual basis [7]. Due to the rarity of giant retroperitoneal lipoma, and the presence of multiple limb lipomata, *HMGA2* gene testing was undertaken to assess for familial multiple lipomatosis, which is a rare disease characterized by multiple lipomas of the trunk and limbs. Its underlying genetic cause is unknown, although literature suggests deregulation of the *HMGA2* gene which encodes for aberrant cell proliferation and development of benign tumours may be responsible [11]. Despite him having multiple superficial lipomas, his testing was negative.

## CONTRIBUTOR

Dr Geoffrey Strutton. Department of Anatomical Pathology, Princess Alexandra Hospital, Woolloongabba, QLD 4102, Australia.

## PATIENT CONSENT

Written informed consent was obtained from the patient for publication of this case report and accompanying images. A copy of the written consent is available for review by the Editor-in-Chief of this journal on request.

## CONFLICT OF INTEREST STATEMENT

None declared.

## FUNDING

None.
